# Nationwide trends in prehospital blood product use after injury 2020–2023

**DOI:** 10.1111/trf.18221

**Published:** 2025-04-04

**Authors:** Christine Carico, Chandler Annesi, N. Clay Mann, Matthew J. Levy, Pawan Acharya, Timothy Hurson, Daniel Lammers, Jan O. Jansen, Jeffrey D. Kerby, John B. Holcomb, Zain G. Hashmi

**Affiliations:** ^1^ Center for Injury Science University of Alabama at Birmingham Birmingham Alabama USA; ^2^ Division of Trauma and Acute Care Surgery, Department of Surgery University of Alabama at Birmingham Birmingham Alabama USA; ^3^ Department of Pediatrics University of Utah School of Medicine, University of Utah Salt Lake City Utah USA; ^4^ Department of Emergency Medicine Johns Hopkins University School of Medicine Baltimore Maryland USA; ^5^ Department of Fire and Rescue Services Office of the Chief Medical Officer Howard County Maryland Mariottsville USA; ^6^ Department of General Surgery The University of North Carolina at Chapel Hill Chapel Hill North Carolina USA

**Keywords:** blood management, transfusion practices (surgical)

## Abstract

**Introduction:**

Prehospital blood transfusion improves survival after injury. Understanding potential demand for and usage of prehospital blood transfusion is important to help improve supply and utilization of this prehospital intervention. The primary objective of this study is to describe potential current demand for prehospital blood product in adults after blunt and penetrating injury from 2020 to 2023. We also estimate the extent to which this potential demand is being met.

**Methods:**

Patients ≥16 years with blunt/penetrating injuries included in the National Emergency Medical Services Information System (NEMSIS) from 2020 to 2023 were identified. Patients were classified into Cohort 1 (systolic blood pressure (SBP) <90 and heart rate (HR) >108 or SBP <70) and Cohort 2 (shock index ≥1), and total numbers in each cohort were reported. Additionally, the number and percentage of patients who were potentially eligible for and who received prehospital blood transfusion were calculated and trended over time.

**Results:**

After exclusions, 20.4 million trauma patients were included. A total of 262,761 Cohort 1 patients and 1,227,556 Cohort 2 patients were potentially eligible for transfusion. Estimated demand for blood transfusion increased from 2020 to 2023 (*p* < 0.001) in both cohorts. Cohort 1 had the highest estimated proportion of patients (0.9%, *n* = 2,289) who received transfusion, demonstrating that few potentially eligible adult trauma patients received blood product.

**Conclusions:**

Altogether, 1.2 million hemodynamically unstable trauma patients were potentially eligible for prehospital blood transfusion after injury during 2020–2023, yet less than 1% received this intervention. These data underscore the need to evaluate and resolve barriers to wider use of prehospital blood transfusions.

**Abbreviations**
EMS: Emergency Medical Services
HR: heart rate
NEMSIS: National Emergency Medical Services Information System
NHTSA: National Highway Traffic Safety Administration
SBP: systolic blood pressure

## 1 INTRODUCTION

Hemorrhage is a leading cause of preventable deaths from traumatic injury.^1^, ^2^, ^3^ Several large studies and a prehospital randomized controlled trial have demonstrated that prehospital administration of blood products has the potential to save lives among patients in traumatic hemorrhagic shock.^4^, ^5^, ^6^, ^7^, ^8^ As a result, contemporary military and civilian clinical practice guidelines now support blood product‐based prehospital resuscitation.^9^

Several initiatives in recent years have attempted to move these guidelines into civilian practice. A 2016 pilot program was launched in Houston, TX, in which ground EMS services carried packed red blood cells (RBCs) and liquid plasma (LP), with several other EMS agencies within Texas and North Carolina following suit over the subsequent 2 years.^10^ In 2017, Low Titer O Positive Whole Blood (LTO + WB) was approved as an Emergency Release Blood Product, allowing ground EMS services to carry this product for prehospital resuscitation of injured patients in San Antonio, TX.^11^, ^12^ We had previously reported the number of patients eligible for prehospital blood product resuscitation based on varying criteria of hemodynamic instability in the United States in 2019 in order to provide a quantifying benchmark for national potential demand for prehospital blood products.^13^ We also quantified the rate of usage of prehospital blood products in adult trauma patients in 2019 that met these criteria of hemodynamic instability to identify how well this demand was met.^13^ Surprisingly, this study found that only 0.4% of potentially eligible adult trauma patients received prehospital blood products, highlighting the need for augmented strategies to increase the availability of prehospital blood products.

Despite these efforts, as of October 2024, it is estimated that only 205 ground EMS agencies have implemented the usage of prehospital blood product in the initial resuscitation of trauma patients (personal communication from Randall Schaefer). Although this is an increase from prior years, it accounts for only 1% of all EMS agencies nationwide.^10^ However, it is unclear if the potential demand for and usage of prehospital blood products has changed since our initial study in 2019, especially as more EMS agencies are now trained and equipped. Elucidating the current climate of potential prehospital blood product demand and relative usage is essential to guide further policy and interventions to augment availability. The primary objective of this study is to determine the overall potential demand for prehospital blood product between the years 2020 and 2023 in the United States. Our secondary objective is to estimate the proportion of trauma patients who received a prehospital blood transfusion among those meeting criteria for hemodynamic instability. Given the continued small proportion of ground EMS agencies carrying blood product, we anticipate that there remains a large unmet need for prehospital blood transfusion.

## 2 METHODS

We conducted a retrospective, longitudinal analysis using the National Emergency Medical Services Information System (NEMSIS) 2020–2023 databases. The NEMSIS databases collect and standardize EMS data from states and territories nationwide for each calendar year.^14^ NEMSIS databases are a product of the National Highway Traffic Safety Administration's (NHTSA) Office of EMS in collaboration with the University of Utah, which serves as the host of the Technical Assistance Center (TAC).^14^ NEMSIS databases include a standardized set of data elements collected by all participating EMS agencies that were uniformly captured over the study period.^15^ Data collection marginally increased over the study period and represented 43 million (in 2020) to 54 million (in 2023) activations per year from 12,000 (2020) to 14,000 (2023) EMS agencies nationwide.^16^, ^17^, ^18^, ^19^

**FIGURE 1:**
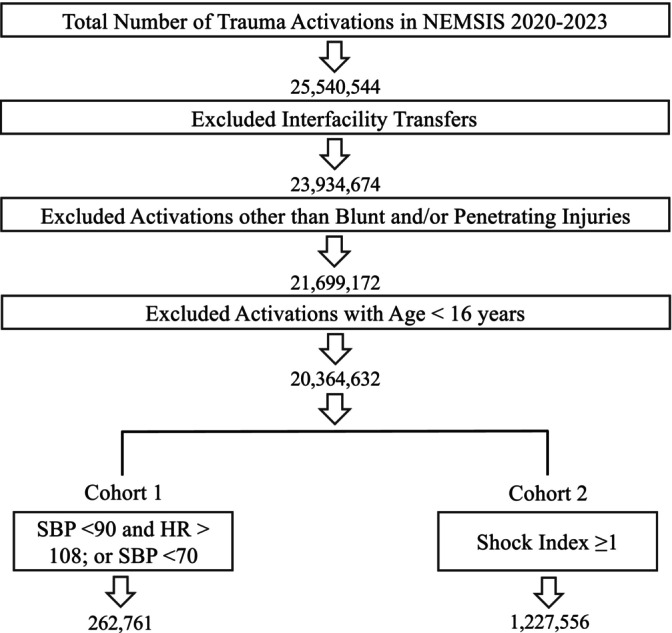
Overview of study population and exclusion criteria. Trauma activations included in the National Emergency Medical Services Information System (NEMSIS) were collected. Exclusion criteria were applied, and the remaining patients were categorized into two groups based on different measures of hemodynamic instability (Cohorts 1 and 2, respectively).

All trauma activations reported to the NEMSIS 2020–2023 databases were identified by selecting for EMS encounters initiated for possible injury (data element eSituation.02 of the NEMSIS databases). EMS activations for interfacility transfers were excluded. EMS air and ground activations involving adult patients (≥16 years old) who experienced blunt and/or penetrating causes of injury were included (Figure 1). Causes of injury corresponding to traumatic injury were identified (i.e., excluding nontraumatic injuries such as foreign body ingestion, burns, drowning, and poisoning) from the NEMSIS suggested code list in ICD‐10‐CM and included in the analysis (Table 1). Non‐transport encounters were also included as non‐transport crews can also supply, and therefore document use of, prehospital blood product (Table 1). Non‐transport administrative refers to the documenting EMS unit's role in coordination, oversight, and/or supervision of EMS services provided at the scene. Non‐transport assistance refers to the documenting EMS unit's role in providing care at the scene, but transport of the patient is ultimately done by another unit. Finally, non‐transport rescue refers to the documenting EMS unit's role in specialized support at the scene (e.g., fire suppression, extrication, technical rescue).^20^

**TABLE 1:** Descriptive statistics of pooled 2020–2023 data extracted from NEMSIS for Cohort 1 and Cohort 2.

		Cohort 1 (SBP <90 and HR >108; or SBP <70)		Cohort 2 (Shock Index ≥1)	
		*N* = 262,761		*N* = 1,227,556	
		Total	%	Total	%
Age	16–25	32,308	12.3	257,605	21.0
	26–35	34,300	13.1	219,046	17.8
	36–45	28,164	10.7	144,808	11.8
	46–55	28,906	11	113,630	9.3
	56–65	40,758	15.5	142,741	11.6
	66–75	42,834	16.3	144,730	11.8
	76–85	34,337	13.1	122,713	10.0
	86–100	18,500	7.0	76,936	6.3
	>100	708	0.3	1,997	0.2
	Missing	1,946	0.7	3,350	0.3
Sex	Female	104,792	39.9	601,767	49.0
	Male	157,083	59.8	622,395	50.7
	Unknown	313	0.1	1,293	0.1
	Missing	573	0.2	2,101	0.2
Cause of injury	Firearm	21,769	8.3	35,464	2.9
	Stab	10,983	4.2	56,967	4.6
	Motor Vehicle Collision	55,965	21.3	289,490	23.6
	Struck by/Blunt Assault	13,213	5.0	149,992	12.2
	Fall	130,042	49.5	562,189	45.8
	Machinery	856	0.3	3,120	0.3
	Pedestrian Struck	7,399	2.8	23,697	1.9
	Animal	1,544	0.6	10,439	0.9
	Pedal Cyclist	1,502	0.6	7,319	0.6
	Other	19,488	7.4	88,879	7.2
Patient acuity	Critical	62,623	23.8	122,649	10.0
	Emergent	66,334	25.2	303,411	24.7
	Lower Acuity	57,131	21.7	480,405	39.1
	Dead without resuscitation	9,684	3.7	440	0.0
	Missing	66,989	25.5	320,651	26.1
Primary role of EMS unit	Ground Transport	224,659	85.5	1,076,753	87.7
	Air Transport	12,042	4.6	28,339	2.3
	Non‐Transport Assistance	10,860	4.1	51,550	4.2
	Non‐Transport Rescue	13,332	5.1	65,901	5.4
	Non‐Transport Administrative	1,863	0.7	5,001	0.4
	Missing	5	0.0	12	0.0
Urbanicity	Rural	21,332	8.1	82,848	6.8
	Suburban	16,917	6.4	69,010	5.6
	Urban	212,756	81.0	1,027,419	83.7
	Wilderness	5,381	2.1	21,173	1.7
	Missing	6,375	2.4	27,106	2.2
Census Division	East North Central	31,675	12.1	144,719	11.8
	East South Central	18,552	7.1	75,371	6.1
	Middle Atlantic	15,068	5.7	66,933	5.5
	Mountain	23,475	8.9	129,545	10.6
	New England	10,876	4.1	44,841	3.7
	Pacific	36,601	13.9	182,441	14.9
	South Atlantic	67,478	25.7	313,002	25.5
	West North Central	18,664	7.1	86,915	7.1
	West South Central	40,357	15.4	183,676	15.0
	Missing	15	0.0	113	0.0

Abbreviation: HR, heart rate; SBP, systolic blood pressure in mmHg.

Patients meeting inclusion criteria were then separated into two cohorts based on established definitions of hemodynamic instability reported in the literature to identify patients that could potentially benefit from prehospital blood product resuscitation. Cohort 1 included patients with a systolic blood pressure (SBP) <90 and heart rate (HR) >108 or SBP <70 alone regardless of HR (Figure 1). This definition of hemodynamic instability has been utilized in several large‐scale prehospital postinjury resuscitative trials, including Prehospital Air Medical Plasma (PAMPer), Control of Major Bleeding After Trauma (COMBAT), Trauma Resuscitation with Low‐Titer Group O Whole Blood or Products (TROOP), and Type O Whole Blood and Assessment of AGE during Prehospital Resuscitation (TOWAR).^6^, ^21^, ^22^, ^23^, ^24^, ^25^, ^26^ Cohort 2 included patients with a calculated shock index of ≥1. Shock index is defined as heart rate divided by systolic blood pressure; values ≥1 have been shown to predict the need for massive transfusion as well as mortality in trauma.^27^, ^28^, ^29^, ^30^ Both definitions are commonly incorporated into prehospital treatment protocols to initiate transfusion. These cohorts were chosen to encompass both conservative (Cohort 1) and more liberal (Cohort 2) definitions of hemodynamic instability. Of note, these cohorts were not designed to be mutually exclusive, and some patients could be included in both cohorts. Inclusion of patients in either or both cohorts by vital sign criteria was based on the single worst value documented during transport.

Patient‐level variables extracted from the database included age, gender, cause of injury, patient acuity on EMS arrival at the scene (critical, emergent, lower acuity, dead without resuscitation), primary role of the EMS unit (ground transport, air transport, non‐transport assistance, non‐transport rescue, non‐transport administrative), incident location (rural, suburban, urban, wilderness), and region of the country (as determined by Census Division) (Tables 1 and S1). The EMS Core Content dictionary was used to define patient acuity upon EMS arrival at the scene. Critical patient acuity indicates that the patient is deemed to have a life‐threatening injury with a high probability of mortality if immediate intervention is not initiated to prevent further airway, breathing, hemodynamic, and/or neurologic instability. Emergent patient acuity indicates that a patient has an injury that may progress in severity or cause high‐morbidity complications if treatment is not initiated quickly. Finally, lower acuity patients are those with injuries that have a low probability of progression to more serious presentations.^31^ Given the potential for incomplete data in NEMSIS, we utilized variables encoded as mandatory and provided a supplementary data table demonstrating the number of entries reported as “missing” or “other” to put in context the amount of missing data in variables we interrogated in this study (Tables 1 and S1).

**FIGURE 2:**
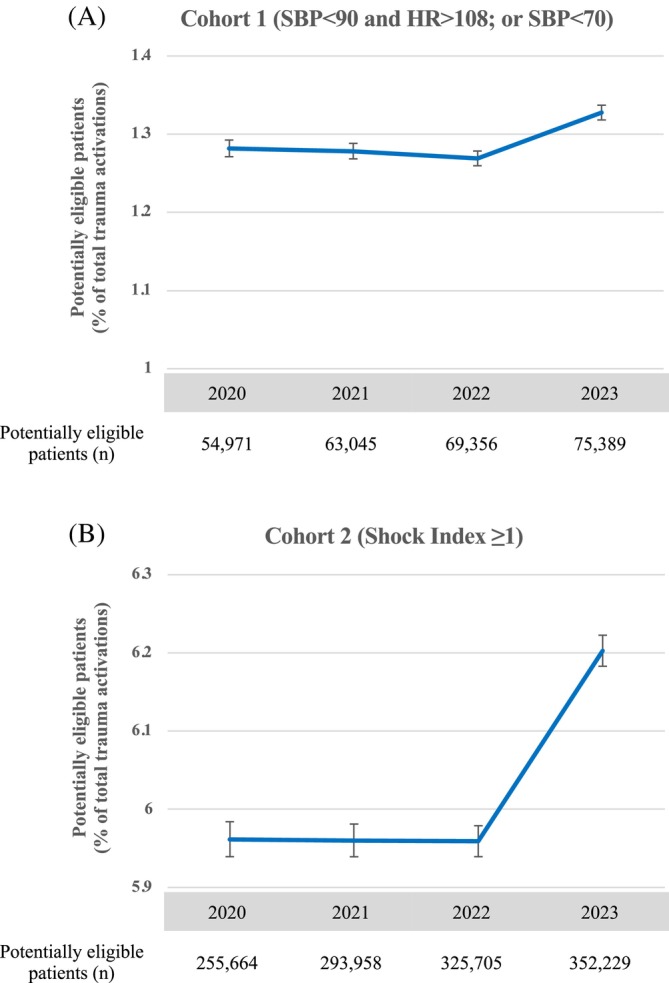
Trend in potential demand for prehospital blood product by year. Adult trauma patients potentially eligible for prehospital blood product reported as a proportion of total number of trauma activations per year for (A) Cohort 1 (SBP < 90 and HR >108; or SBP < 70), and (B) Cohort 2 (Shock Index ≥1) with 95% confidence intervals plotted for each year. Total number of potentially eligible adult trauma patients per year reported below each graph.

**FIGURE 3:**
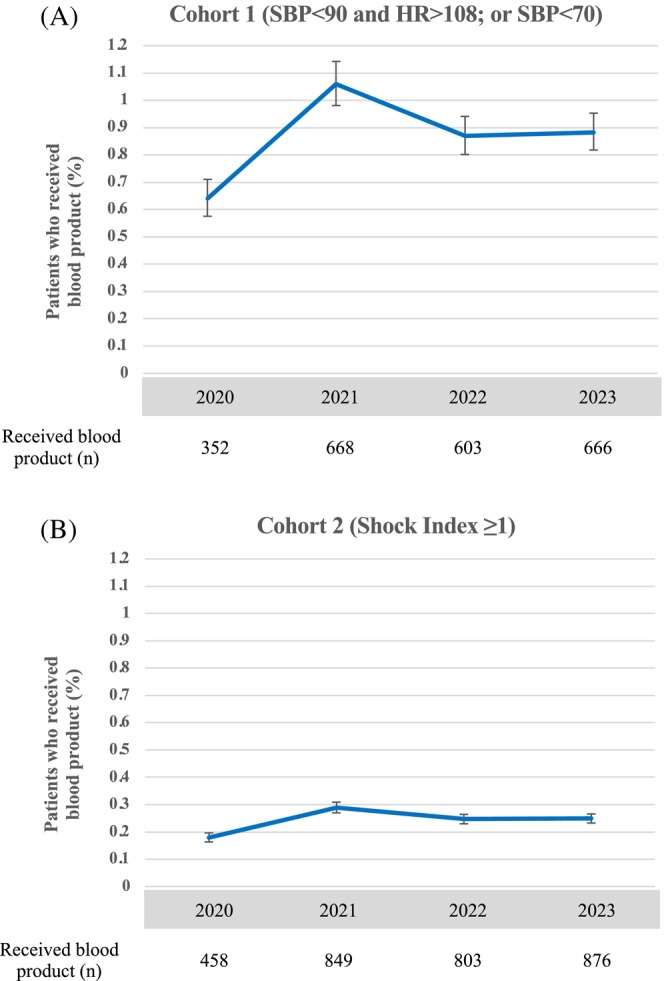
Trend in usage of prehospital blood product by year. Percentage of total potentially eligible adult trauma patients who received prehospital blood product reported for A) Cohort 1 (SBP < 90 and HR > 108; or SBP < 70), and (B) Cohort 2 (Shock Index ≥1) with 95% confidence intervals plotted for each year. Total number of potentially eligible adult trauma patients who received prehospital blood product per year reported below each graph.

In addition to identifying patients who could potentially benefit from prehospital blood products, we also identified documented prehospital blood product utilization (of whole blood as well as component products) in both cohorts of patients to determine the percentage of hemodynamically unstable patients that received prehospital blood product resuscitation. Usage of prehospital blood products was identified using medication codes corresponding to respective blood product transfusions based on NEMSIS coding. Data from each year of this study was extracted and initially analyzed separately to determine trends in potential prehospital blood product need and usage by year from 2020 to 2023 (Figures 2 and 3, Tables S2 and S3). Since motor vehicle collision (MVC) remains a leading cause of injury‐related mortality in the United States, we report results for potential blood product need in this cohort separately.^32^ Data was then pooled to give total numbers and percentages over the study period (Table 2).

**TABLE 2:** National estimates of potential need for prehospital blood product and current prehospital blood utilization among adult trauma patients in the United States reported to NEMSIS 2020–2023.

	Cohort 1		Cohort 2	
	SBP <90 and HR >108; or SBP <70		Shock index ≥1	
	*N* = 262,761		*N* = 1,227,556	
Estimated need for prehospital blood product, units^a^	262,761		1,227,556	
Current prehospital blood utilization^b^	*n*	%	*n*	%
Any blood product	2,289	0.87	2,986	0.24
Blood product “not otherwise specified”	2,118	0.81	2,698	0.22
Packed red blood cells	65	0.02	79	0.01
Plasma	29	0.01	27	0.002
Platelet	0	0.00	0	0.00
Cryoprecipitate	34	0.01	100	0.01
Coagulation factor	0	0.00	0	0.00
Whole blood	57	0.02	98	0.01

Abbreviations: HR, heart rate; SBP, systolic blood pressure in mmHg.

^a^ Based on prior data, the average amount of blood transfused among adult trauma patients in the prehospital phase is estimated to be one unit of whole blood or one unit of packed red blood cells and one unit of plasma.

^b^ Reflects number (%) of activations where blood products were utilized and not necessarily the number of units transfused.

Data coded in NEMSIS databases does not reflect the number of units of blood product transfused per patient but rather reflects a binary variable indicating if there was a blood product transfusion or not. As in our prior study, the national estimate of the potential need (i.e., demand) for prehospital blood was calculated by multiplying the number of patients in each cohort described above with the median number of blood products used in a prehospital setting for trauma resuscitation.^13^ This was deemed to be the equivalent of one unit of whole blood based on blood products utilized in the Multicenter Observational Prehospital Resuscitation on Helicopter Study (PROHS) (one unit of plasma and one unit of pRBCs, similar to one unit of whole blood in terms of volume and intended effect^33^) and PAMPer trial (two units of plasma in the intervention arm vs. two units of pRBCs in the standard of care arm, both similar to whole blood in terms of volume).^6^, ^34^

We estimated the proportion and 95% confidence interval (CI) in blood product potential demand and utilization and assessed the trend using the Cochran‐Armitage Trend Test^35^ (Tables S2 and S3). Statistical significance was set at two‐sided α <0.05. We followed STROBE guidelines for cross‐sectional studies for reporting this study (File S4). All analysis was performed using Stata16/SE statistical software package (StatCorp, College Station, TX).

## 3 RESULTS

In total, there were 25.5 million trauma activations reported from 2020 to 2023 in NEMSIS. After applying the inclusion and exclusion criteria, 20.4 million activations remained for analysis (Figure 1). Patients who could potentially benefit from prehospital blood product based on hemodynamic instability ranged from 262,761 in Cohort 1 (SBP < 90 and HR > 108 or SBP < 70 alone irrespective of HR) to 1,227,556 in Cohort 2 (shock index ≥1) (Figure 1, Table 2). Of the 262,761 total trauma patients in Cohort 1, 32,752 (12.5%) were patients with penetrating injury and 230,009 (87.5%) were patients with blunt injury (Table 1, Table S1). Of the 1,227,556 total trauma patients in Cohort 2, 92,431 (7.5%) were patients with penetrating injury and 1,135,125 (92.5%) were patients with blunt injury (Tables 1 and S1). Among Cohort 1 patients with blunt injury, 55,965 (21.3%) were injured in an MVC, and among Cohort 2 patients with blunt injury, 289,490 (23.6%) were injured in an MVC (Tables 1 and S1). Other mechanisms of injury included blunt assault, fall, machinery, pedestrian struck, animal, pedal cyclist, and otherwise not defined (Tables 1 and S1). Patients whose clinical status was identified as “critical” ranged from 62,623 (23.8%) in Cohort 1 to 122,649 (1%) in Cohort 2 (Tables 1 and S1).

Only between 0.87% (Cohort 1) and 0.24% (Cohort 2) of potentially eligible patients received a prehospital blood transfusion from 2020 to 2023 (Table 2). We also analyzed each year separately to determine the number of patients potentially eligible for prehospital blood product and how many received prehospital blood product by year (Figures 2 and 3, Tables S2 and S3). In Cohort 1, there were 54,971 (1.28% of total trauma activations; 95% CI: 1.27%–1.29%) potentially eligible patients in 2020 and 75,389 (1.33% of total trauma activations, 95% CI: 1.32%–1.34%) potentially eligible patients by 2023, with a statistically significant increase between 2020 and 2023 (*p* < 0.001) (Figure 2, Table S2). In Cohort 2, there were 255,664 (5.96% of total trauma activations; 95% CI: 5.94%–5.98%) potentially eligible patients in 2020 and 352,229 (6.20% of total trauma activations; 95% confidence interval: 6.18%–6.22%) potentially eligible patients by 2023, with a statistically significant increase between 2020 and 2023 (*p* < 0.001) (Figure 2, Table S2). Usage of prehospital blood product increased between 2020 and 2023 from 0.64% (95% CI: 0.58%–0.71%) to 0.88% (95% CI: 0.82%–0.95%) in Cohort 1 and from 0.18% (95% CI: 0.16%–0.20%) to 0.25% (95% CI: 0.23%–0.27%) in Cohort 2 (*p* < 0.001). However, this represents only a marginal increase, and overall blood product usage remained low over the study period (Figure 3, Table S3).

## 4 DISCUSSION

In this study, we show that 260,000 to as many as 1.2 million patients could have potentially benefitted from prehospital blood resuscitation after traumatic injury between the years 2020–2023. The number of patients meeting criteria for requiring a prehospital blood transfusion increased in both cohorts over the study duration. Despite the increasing need, less than 1% of potentially eligible trauma patients were documented to have received any prehospital blood transfusion. The results of this study highlight both the high potential demand and unmet need for prehospital blood products.

These findings provide longitudinal evidence corroborating our prior study.^13^ This report demonstrated that in 2019 as many as 300,000 trauma patients could potentially benefit from prehospital transfusion by a cohort‐matched definition of hemodynamic instability (i.e., shock index ≥1).^13^ By 2023 this number had increased to 350,000. These data show that the potential demand for prehospital blood remains high and continues to increase. Prior independent studies conducted at a regional level have also consistently shown high potential demand for prehospital transfusion.^1^, ^3^, ^36^, ^37^, ^38^ For example, in a 2014 a multicenter retrospective study in Harris County, Texas revealed that 34.5% of trauma deaths within 1 h of arrival at the hospital were due to potentially preventable hemorrhage.^37^ This high potential demand for prehospital blood product highlights the critical impact of trauma, particularly hemorrhage, as a leading cause of death in the United States, underscoring the urgent need for expanding existing systems of care to meet this growing challenge.^1^, ^2^, ^3^, ^32^, ^37^

Despite this high and increasing potential demand, less than 1% of eligible trauma patients received this lifesaving intervention. This utilization was only a nominal increase from 2019, in which we showed that approximately 0.4% of potentially eligible trauma patients received prehospital blood. There is a paucity of studies quantifying usage of prehospital blood product at the national level in the United States, with the only other study reporting similar results.^39^ However, indirect evidence from studies reporting the number of potentially preventable deaths due to hemorrhage supports our findings of an unmet need for prehospital blood.^1^, ^37^, ^38^ This reported unmet potential need represents either underutilization or underdocumentation of use, or a combination of both. There are several factors that likely contribute to underutilization of prehospital blood product. First, there is no standardized indication for the need for prehospital blood transfusion, so utilization is at the discretion of each EMS agency. A prior meta‐analysis of 71 studies showed that there was no singular criterion for initiation of transfusion, but common transfusion triggers included hypotension, tachycardia, traumatic cardiac arrest, signs of poor tissue perfusion, type of injury, and, finally, clinician judgment.^40^, ^41^, ^42^ Additionally, while there are national guidelines dictating recommendations for the scope of practice of prehospital clinicians, each state is autonomous in determining the scope of practice for prehospital emergency providers. In most states, paramedics may initiate transfusions, while in others it is required for a nurse or physician‐level provider to be present.^10^ Additionally, despite efforts to increase access to prehospital blood products, only 1% of EMS agencies carry blood products.^10^, ^43^ This is likely a reflection of inadequate supply and prohibitive expense, with per‐patient cost of up to $1000 per transfusion.^44^, ^45^ This low utilization, yet high potential demand, highlights the significant work that must be done for availability and subsequent usage to increase.

We recognize and acknowledge several limitations of this analysis. The foremost limitation we acknowledge is the potential undercounting of blood product utilization through documentation in the NEMSIS database. The current EMS reimbursement mechanism does not incorporate prehospital transfusion, providing no incentive for documentation of this intervention by EMS providers. Similarly, submissions to NEMSIS are made voluntarily, which can potentially introduce selection bias. However, given that our results are consistent with reports that suggest that only 1% of agencies nationwide have access to prehospital blood, we do not believe this is a large‐scale underestimation that negates this study's findings. We also acknowledge there may be potential overreporting of demand for prehospital blood product due to the use of a single worst documented vital sign. Inclusion criteria used as a proxy for hemorrhagic shock after traumatic injury were based on a single maximum HR and a single minimum SBP value recorded during the encounter. Therefore, there is a potential that some of these values were either recorded erroneously or in patients with only a brief period of hemodynamic instability. However, our inclusion criteria based on worst vital sign recording is consistent with the TOWAR trial protocol,^24^ which defines enrollment eligibility as the moment at which the patient meets the vital sign threshold. Therefore, within these limitations, we are confident in our reporting of the significant nationwide potential demand for prehospital blood product in adult trauma patients.

As hemorrhage is the most common cause of potentially preventable death from trauma, and evidence suggests that prehospital blood transfusion could reduce mortality after traumatic injury,^4^, ^5^, ^6^, ^7^, ^8^, ^34^, ^46^ these data have strong implications for the necessity of improved blood product delivery in the prehospital setting. There are several barriers to the widespread usage of prehospital blood products. Perhaps the most significant barrier is adequate supply. Our data show that up to 1.2 million units of blood were required over a four‐year period to meet the demand for the prehospital needs of traumatically injured patients, averaging 300,000 per year, and this number continues to increase. This number is in addition to the blood products required for the prehospital management of nontraumatic hemorrhage (e.g., GI bleed) as well as in‐hospital transfusion requirements. As of October 2024, prehospital blood is supplied by independent regional blood suppliers or directly from inventory from a hospital's transfusion service, placing a disproportional burden on regions with high need but low population.^10^ Reimbursement is another major barrier, given the cost of blood products as discussed above. A recent survey was administered to all ground EMS agencies with an active blood transfusion program and found that only 11% report receiving reimbursement for this costly intervention.^47^ Not only is this a major barrier overall to implementation given the high cost of blood products, but one that will disproportionately affect rural‐based and volunteer EMS agencies in underserved areas.^44^

Overall, this study highlights two findings: that nationally there is high potential demand for prehospital blood product in adult patients experiencing blunt and penetrating trauma, and that much of this potential demand remains unmet. As it has been shown that prehospital blood product could potentially reduce preventable death in patients with traumatic injury, coordinated effort from multiple stakeholders is needed to circumvent barriers and improve utilization of this prehospital intervention on a national scale. This will require collaborative effort between healthcare agencies, blood suppliers, providers, policy makers, and payers responsible for reimbursement, with the ultimate goal of reducing preventable deaths due to traumatic hemorrhage.

## CONFLICT OF INTEREST STATEMENT

M.J.L is the noncompensated Chairperson of the nonprofit Stop the Bleed Coalition. M.J.L. is a consultant for Stryker Medical Education.

## Supporting information


**Table S1.** Descriptive statistics of 2020–2023 data by year extracted from NEMSIS for Cohort 1 and Cohort 2.

Table S2. Adult trauma patients potentially eligible for prehospital blood product by year.

Table S3. Adult trauma patients who received prehospital blood product by year.

**Data S1.** Supporting Information.

## References

[1] Davis JS , Satahoo SS , Butler FK , Dermer H , Naranjo D , Julien K , et al. An analysis of prehospital deaths: who can we save? J Trauma Acute Care Surg. 2014;77(2):213–218. 25058244 10.1097/TA.0000000000000292

[2] Tisherman SA , Schmicker RH , Brasel KJ , Bulger EM , Kerby JD , Minei JP , et al. Detailed description of all deaths in both the shock and traumatic brain injury hypertonic saline trials of the resuscitation outcomes consortium. Ann Surg. 2015;261(3):586–590. 25072443 10.1097/SLA.0000000000000837 PMC4309746

[3] Eastridge BJ , Holcomb JB , Shackelford S . Outcomes of traumatic hemorrhagic shock and the epidemiology of preventable death from injury. Transfusion. 2019;59(S2):1423–1428. 30980749 10.1111/trf.15161

[4] Holcomb JB , del Junco DJ , Fox EE , Wade CE , Cohen MJ , Schreiber MA , et al. The prospective, observational, multicenter, major trauma transfusion (PROMMTT) study: comparative effectiveness of a time‐varying treatment with competing risks. JAMA Surg. 2013;148(2):127–136. 23560283 10.1001/2013.jamasurg.387 PMC3740072

[5] Holcomb JB , Tilley BC , Baraniuk S , Fox EE , Wade CE , Podbielski JM , et al. Transfusion of plasma, platelets, and red blood cells in a 1:1:1 vs a 1:1:2 ratio and mortality in patients with severe trauma: the PROPPR randomized clinical trial. JAMA. 2015;313(5):471–482. 10.1001/jama.2015.12 25647203 PMC4374744

[6] Sperry JL , Guyette FX , Brown JB , Yazer MH , Triulzi DJ , Early‐Young BJ , et al. Prehospital plasma during air medical transport in trauma patients at risk for hemorrhagic shock. N Engl J Med. 2018;379(4):315–326. 30044935 10.1056/NEJMoa1802345

[7] Shackelford SA , del Junco D , Powell‐Dunford N , Mazuchowski EL , Howard JT , Kotwal RS , et al. Association of Prehospital Blood Product Transfusion during Medical Evacuation of combat casualties in Afghanistan with acute and 30‐day survival. JAMA. 2017;318(16):1581–1591. 29067429 10.1001/jama.2017.15097 PMC5818807

[8] Rehn M , Weaver A , Brohi K , Eshelby S , Green L , Røislien J , et al. Effect of prehospital red blood cell transfusion on mortality and time of death in civilian trauma patients. Shock. 2019;51(3):284–288. 29664833 10.1097/SHK.0000000000001166

[9] Cannon JW , Khan MA , Raja AS , Cohen MJ , Como JJ , Cotton BA , et al. Damage control resuscitation in patients with severe traumatic hemorrhage: a practice management guideline from the eastern Association for the Surgery of trauma. J Trauma Acute Care Surg. 2017;82(3):605–617. 28225743 10.1097/TA.0000000000001333

[10] Schaefer RM , Bank EA , Krohmer JR , Haskell A , Taylor AL , Jenkins DH , et al. Removing the barriers to prehospital blood: a roadmap to success. J Trauma Acute Care Surg. 2024;97(2S Suppl 1):S138–S144. 38689393 10.1097/TA.0000000000004378

[11] Schaefer R , Long T , Wampler D , Summers R , Epley E , Waltman E , et al. Operationalizing the deployment of low‐titer O‐positive whole blood within a regional trauma system. Mil Med. 2021;186(Suppl 1):391–399. 10.1093/milmed/usaa283 33499434

[12] Pokorny DMBMAEPMBDMZCSWCJSRMACE . The use of prehospital blood products in the resuscitation of trauma patients: a review of prehospital transfusion practices and a description of our regional whole blood program in San Antonio, TX. ISBT Sci Ser. 2019;14(3):332–342.

[13] Hashmi ZG , Jansen JO , Kerby JD , Holcomb JB . Nationwide estimates of the need for prehospital blood products after injury. Transfusion. 2022;62(Suppl 1):S203–S210. 35753065 10.1111/trf.16991

[14] Administration, NHTS . National Emergency Medical Services Information System (NEMSIS). December 22, 2024; Available from: https://nemsis.org/.

[15] TAC, N.T.A.C . National EMS Database NEMSIS Public Release Research Data Set v3.4.0 2023 User Manual. 2024 February 20, 2025; Available from: https://nemsis.org/wp-content/uploads/2024/04/2023-NEMSIS-RDS-340-User-Manual.pdf.

[16] NEMSIS . 2020 Public‐Release Research Dataset Announcement . 2020, [February 20, 2025]; Available from: https://nemsis.org/2020-public-release-dataset-now-available/.

[17] NEMSIS . 2021 Public‐Release Research Dataset Announcement . 2021, [February 20, 2025]; Available from: https://nemsis.org/2021-public-release-dataset-now-available/.

[18] NEMSIS . 2022 Public‐Release Research Dataset Flyer . 2022, [February 20, 2025]; Available from: https://nemsis.org/wp-content/uploads/2023/04/Press-Release-NEMSIS-2022-Public-Release-Dataset-4-18-23.pdf.

[19] NEMSIS . 2023 Public‐Release Research Dataset Flyer . 2023, [February 20, 2025]; Available from: https://nemsis.org/wp-content/uploads/2024/04/NEMSIS-2023-Dataset-Flyer.pdf.

[20] TAC (N.T.A.C.) . Extended Data Definitions NEMSIS Version 3.4.0 . February 19, 2025. Available from: https://nemsis.org/wp-content/uploads/2018/09/Extended-Data-Definitions_v3_Final.pdf.

[21] Moore HB , Moore EE , Chapman MP , McVaney K , Bryskiewicz G , Blechar R , et al. Plasma‐first resuscitation to treat haemorrhagic shock during emergency ground transportation in an urban area: a randomised trial. Lancet. 2018;392(10144):283–291. 30032977 10.1016/S0140-6736(18)31553-8 PMC6284829

[22] Bulger EM , May S , Kerby JD , Emerson S , Stiell IG , Schreiber MA , et al. Out‐of‐hospital hypertonic resuscitation after traumatic hypovolemic shock: a randomized, placebo controlled trial. Ann Surg. 2011;253(3):431–441. 21178763 10.1097/SLA.0b013e3181fcdb22 PMC3232054

[23] H.E., J.J.O.W. . *Trauma Resuscitation With Low‐Titer Group O Whole Blood or Products (TROOP)* . ClinicalTrials.gov identifier: NCT05638581. Updated 03‐25‐2024; Accessed 03‐30‐2025. Available from: https://clinicaltrials.gov/study/NCT05638581.

[24] Sperry J. Type O *Whole Blood and Assessment of AGE during Prehospital Resuscitation Trial (TOWAR trial)*. ClinicalTrials.gov identifier: NCT04684719. Updated: 03‐25‐2025; Accessed: 03‐30‐2025. Available from: https://clinicaltrials.gov/study/NCT04684719.

[25] Sperry J. *PreHospital Air Medical Plasma Trial (PAMPer),* ClinicalTrials.gov identifier: NCT01818427. Updated: 04‐01‐2021; Accessed: 03‐30‐2025. Available from: https://clinicaltrials.gov/study/NCT01818427.

[26] Chapman MP , Moore EE , Chin TL , Ghasabyan A , Chandler J , Stringham J , Gonzalez E , Moore HB , Banerjee A , Silliman CC , Sauaia A . Combat: Initial Experience with a Randomized Clinical Trial of Plasma‐Based Resuscitation in the Field for Traumatic Hemorrhagic Shock. Shock. 2015;44 Suppl 1(0 1):63–70. 25784527 10.1097/SHK.0000000000000376 PMC4504796

[27] Zhu CS , Cobb D , Jonas RB , Pokorny D , Rani M , Cotner‐Pouncy T , et al. Shock index and pulse pressure as triggers for massive transfusion. J Trauma Acute Care Surg. 2019;87(1S Suppl 1):S159–S164. 31246921 10.1097/TA.0000000000002333

[28] Vandromme MJ , Griffin RL , Kerby JD , McGwin G Jr , Rue LW III , Weinberg JA . Identifying risk for massive transfusion in the relatively normotensive patient: utility of the prehospital shock index. J Trauma. 2011;70(2):384–388. discussion 388–90. 21307738 10.1097/TA.0b013e3182095a0a

[29] Jehan F , Con J , McIntyre M , Khan M , Azim A , Prabhakaran K , et al. Pre‐hospital shock index correlates with transfusion, resource utilization and mortality; the role of patient first vitals. Am J Surg. 2019;218(6):1169–1174. 31540684 10.1016/j.amjsurg.2019.08.028

[30] El‐Menyar A , Goyal P , Tilley E , Latifi R . The clinical utility of shock index to predict the need for blood transfusion and outcomes in trauma. J Surg Res. 2018;227:52–59. 29804862 10.1016/j.jss.2018.02.013

[31] Administration, NHTS . National EMS Core Content . February 24, 2025; Available from: Table:///Users/christinecarico/Downloads/National_EMS_Core_Content.pdf.

[32] CDC . Injury and Violence Are Leading Causes of Death . 2022 [cited 2024 11 Nov]; Available from: https://wisqars.cdc.gov/animated-leading-causes/.

[33] Nguyen AD , Dasgupta A , Wahed A . Chapter 2 ‐ blood Bank testing and blood products. Management of Hemostasis and Coagulopathies for surgical and critically ill patients. Philadelphia; Elsevier Science: 2016.

[34] Holcomb JB , Swartz MD , DeSantis SM , Greene TJ , Fox EE , Stein DM , et al. Multicenter observational prehospital resuscitation on helicopter study. J Trauma Acute Care Surg. 2017;83(1 Suppl 1):S83–S91. 10.1097/TA.0000000000001484 28383476 PMC5562146

[35] Buonaccorsi JP , Laake P , Veierod MB . On the power of the Cochran‐Armitage test for trend in the presence of misclassification. Stat Methods Med Res. 2014;23(3):218–243. 21878460 10.1177/0962280211406424

[36] Irfan A , Juneja K , Abraham P , Smedley WA , Stephens SW , Griffin RL , et al. Advanced prehospital resuscitative care: can we identify trauma patients who might benefit? J Trauma Acute Care Surg. 2021;91(3):514–520. 33990533 10.1097/TA.0000000000003277

[37] Kalkwarf KJ , Drake SA , Yang Y , Thetford C , Myers L , Brock M , et al. Bleeding to death in a big city: an analysis of all trauma deaths from hemorrhage in a metropolitan area during 1 year. J Trauma Acute Care Surg. 2020;89(4):716–722. 32590562 10.1097/TA.0000000000002833

[38] Carroll SL , Dye DW , Smedley WA , Stephens SW , Reiff DA , Kerby JD , et al. Early and prehospital trauma deaths: who might benefit from advanced resuscitative care? J Trauma Acute Care Surg. 2020;88(6):776–782. 32176169 10.1097/TA.0000000000002657

[39] Hashmi ZG , Chehab M , Nathens AB , Joseph B , Bank EA , Jansen JO , et al. Whole truths but half the blood: addressing the gap between the evidence and practice of pre‐hospital and in‐hospital blood product use for trauma resuscitation. Transfusion. 2021;61(Suppl 1):S348–S353. 34086349 10.1111/trf.16515

[40] van Turenhout EC , Bossers SM , Loer SA , Giannakopoulos GF , Schwarte LA , Schober P . Pre‐hospital transfusion of red blood cells. Part 1: a scoping review of current practice and transfusion triggers. Transfus Med. 2020;30(2):86–105. 10.1111/tme.12667 32080942 PMC7317877

[41] Jama T , Lefering R , Lauronen J , Handolin L . Factors affecting physicians' decision to start prehospital blood product transfusion in blunt trauma patients: a cohort study of Helsinki trauma registry. Transfusion. 2024;64(Suppl 2):S167–S173. 38511866 10.1111/trf.17791

[42] Turnbull C , Clegg L , Santhakumar A , Micalos PS . Blood product Administration in the Prehospital Setting: a scoping review. Prehosp Emerg Care. 2024;1–14. 10.1080/10903127.2024.2386007 39159401

[43] Holcomb JB , Butler FK , Schreiber MA , Taylor AL , Riggs LE , Krohmer JR , et al. Making blood immediately available in emergencies. Transfusion. 2024;64(8):1543–1550. 39031029 10.1111/trf.17929

[44] Coyle C , Zitek T , Pepe PE , Stotsenburg M , Scheppke KA , Antevy P , et al. The implementation of a prehospital whole blood transfusion program and Early results. Prehosp Disaster Med. 2023;38(4):513–517. 37357937 10.1017/S1049023X23005952

[45] Levy MJ , Garfinkel EM , May R , Cohn E , Tillett Z , Wend C , et al. Implementation of a prehospital whole blood program: lessons learned. J Am Coll Emerg Physicians Open. 2024;5(2):e13142. 38524357 10.1002/emp2.13142 PMC10958095

[46] Bulger EM , Jurkovich GJ , Nathens AB , Copass MK , Hanson S , Cooper C , et al. Hypertonic resuscitation of hypovolemic shock after blunt trauma: a randomized controlled trial. Arch Surg. 2008;143(2):139–148; discussion 149. 18283138 10.1001/archsurg.2007.41

[47] Hurson T , Schaefer R , Carico C , Griffin R , Bank E , Krohmer J , Jenkins D , Holcomb J , Hashmi Z . Evaluating Reimbursement for prehospital blood tranfusions: A nationwide survey. Transfusion. 2025;65(Suppl. 1):S6–13. 10.1111/trf.18217 PMC12035981 40150955

